# The impact of returning a pet to the shelter on future animal adoptions

**DOI:** 10.1038/s41598-022-05101-5

**Published:** 2022-01-21

**Authors:** Lauren Powell, Chelsea L. Reinhard, Donya Satriale, Margaret Morris, James Serpell, Brittany Watson

**Affiliations:** 1 School of Veterinary Medicine, University of Pennsylvania, Philadelphia, PA USA; 2 Charleston Animal Society, North Charleston, SC USA

**Keywords:** Human behaviour, Animal behaviour

## Abstract

Unsuccessful animal adoptions are stressful for many owners and may reduce their willingness to adopt again. The goal of this study was to determine the proportion of return owners who adopted post-return and investigate return characteristics that affected the likelihood of post-return adoption. We analyzed adoption records from a South Carolina animal shelter between 2015 and 2019 (*n* = 1999) using a logistic regression model including post-return adoption (binary) and return reason, species, animal sex and age. We found one in 10 individuals adopted from the shelter within 12 months of return, and post-return adoption was associated with return reason and species. Returns due to owner-related reasons, such as the owner’s health (OR 0.20, 95% CI 0.07, 0.57) or unrealistic expectations (OR 0.42, 95% CI 0.19, 0.94) were associated with significantly lower odds of post-return adoption. Owners who returned due to the animal’s health exhibited four times greater odds of post-return adoption compared with behavioral returns (OR 4.20, 95% CI 2.37, 7.45). Our findings highlight the value of ensuring adopters’ expectations are aligned with the reality of ownership and minimizing adopter-animal behavioral incompatibility as unsuccessful animal adoptions can reduce the owner’s willingness to adopt again and may affect the adopter’s relationship with the shelter.

## Introduction

Pet ownership is popular in the United States with 57% of U.S. households estimated to own a pet. Dogs are the most common companion animal and can be found in 38% of U.S. households, while 25% of U.S. households are cat owners^1^. Approximately 3.2 million animals are adopted from animal shelters each year, and recent reports suggest adoption from an animal shelter is typically the preferred method of pet acquisition among prospective owners^2–4^. Garrison and Weiss^4^ found more than 80% of prospective dog owners considered acquiring their dog from a shelter. However, animals are returned to the shelter following adoption in 7% to 20% of all adoptions^5–11^. Returns occur for a variety of reasons, although animal behavior has been consistently documented as a primary cause of return for both dogs and cats^6–8,11,12^. Incompatibility with existing pets and owner’s health concerns, particularly allergies among cat adopters, also lead to a significant number of returned adoptions^11–13^.

A considerable body of research has investigated the effects of the shelter environment on animal health and wellbeing^14–18^, but the effects of pet relinquishment on owner wellbeing has received less scientific attention. In a study of relinquishing owners, DiGiacomo et al.^19^ reported all participants found the decision to surrender their pet very difficult. In the only study to date to investigate owners’ perceptions of returning a newly adopted animal, Shore^20^ found most owners thought the experience was very difficult. Forty-one percent of returning owners indicated they would not adopt a pet in the future and a further 13% were unsure whether they would adopt again^20^. These data suggest unsuccessful animal adoptions may detrimentally affect individuals’ desire to own a companion animal in the future, although no empirical evidence exists to support this hypothesis.

The unsuccessful animal adoption experience is likely to vary for each human-animal dyad which could differentially affect the likelihood of owners adopting again post-return. For example, Shore^20^ found that some returning owners indicated they would not adopt again—e.g., one adopter whose child was allergic to the pet—while others indicated they would adopt a different animal in the future, such as an adopter whose landlord had a pet weight limit which prevented her from keeping the pet^20^. To our knowledge, the effect of return characteristics on the odds of post-return adoptions has not yet been investigated. The aims of this study were to investigate the rate of post-return adoptions at a large animal shelter in the Southeastern United States, and to determine whether characteristics of unsuccessful animal adoptions affect the likelihood of post-return adoptions.

## Results

**Figure 1:**
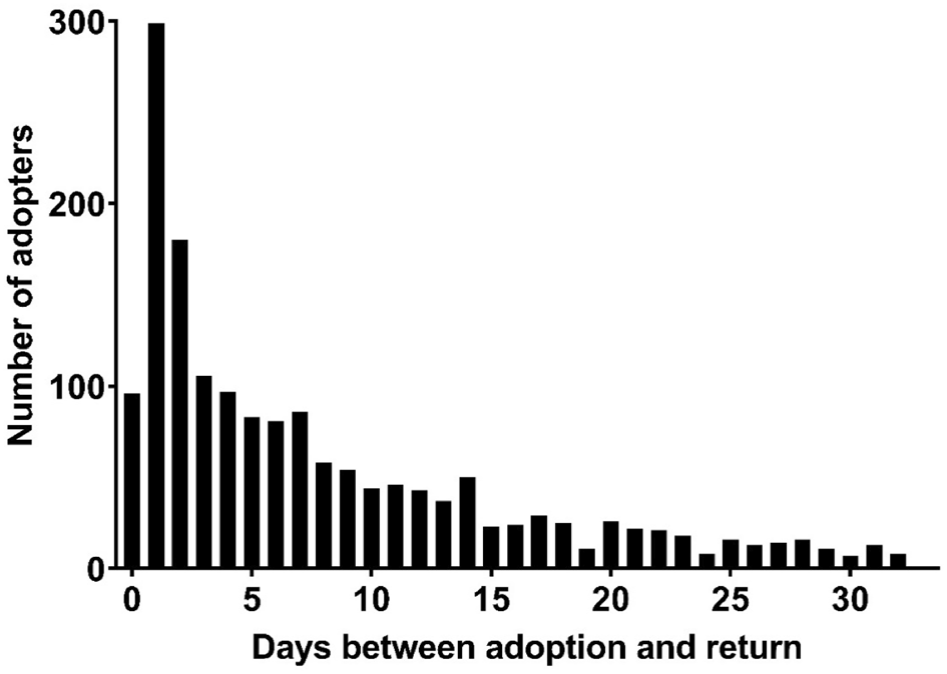
Length of adoption (days) for animals returned within 1 month (*n* = 1666).

A 5-year retrospective analysis of adoption records from Charleston Animal Society (South Carolina, USA) showed 1999 owners returned animals to the shelter following adoption, giving an overall return rate of 9.2%^11^. The vast majority of individuals returned one animal (*n* = 1899, 95.0%), although 4.4% returned two animals (*n* = 88), 10 adopters returned three animals and two adopters returned four animals. Individuals who returned more than one animal during the study period were excluded from further analyses (*n* = 100). Cases where more than two animals were adopted prior to return were also excluded from the logistic regression models (*n* = 214). The returned animals included 1486 dogs, 402 cats, nine rabbits and two barnyard animals. The mean length of ownership was 8.68 days (SD 16.70, Fig. 1).

The characteristics of the returned animals, the reasons for return and the animals’ outcomes post-return have been described in detail elsewhere^11^. In brief, most returned animals were adults (38.2%), 28.6% were young adults, 14.1% were under the age of 6 months and 3.8% were seniors. The majority of returns were also male (54.9%). Behavior was the most common return reason accounting for 32.8% of returns, followed by incompatibility with existing pets at 21.5% and owner circumstances at 11.2%. Return reasons differed between dogs and cats (*X*_2_ = 138.54, p < 0.001). Post-hoc analyses using standardized residuals showed dogs were returned more frequently than cats for behavior (36.1%) and housing issues (11.3%), and cats were returned more due to the health of the owner (17.8%) and the health of the animal (9.8%). The detailed return reasons are provided in Table 1.

**Table 1:** Reasons for returned adoptions of cats and dogs by readoption status (≤ 12 months), excluding cases where more than one animal was returned, or two or more animals were adopted prior to return (*n* = 1675).

Return reason	No readoption				Readoption				Total			
	Dog		Cat		Dog		Cat		Dog		Cat	
	*n*	%	*n*	%	*n*	%	*n*	%	*n*	%	*n*	%
**Behavior**	437	35.6	54	19.0	45	41.7	18	33.3	482	36.1	72	21.3
Aggression to animals	51	4.1	5	1.8	11	10.2	1	1.9	62	4.6	6	1.8
Aggression to people	17	1.4	3	1.1	3	2.8	1	1.9	20	1.5	4	1.2
Behavior issues (multiple)	146	11.9	21	7.4	16	14.8	7	13.0	162	12.1	28	8.3
Bite history	15	1.2	1	0.4	2	1.9	1	1.9	17	1.3	2	0.6
Chases animals	8	0.7	–	–	1	0.9	–	–	9	0.7	–	–
Destructive	49	4.0	1	0.4	3	2.8	1	1.9	52	3.9	2	0.6
Escapes	14	1.1	1	0.4	1	0.9	–	–	15	1.1	1	0.3
Needs too much attention	20	1.6	2	0.7	–	–	1	1.9	20	1.5	3	0.9
Not friendly	–	–	1	0.4	–	–	1	1.9	–	–	2	0.6
Not housebroken/house soiling	13	1.1	4	1.4	–	–	1	1.9	13	1.0	5	1.5
Temperament	7	0.6	2	0.7	1	0.9	2	3.7	8	0.6	4	1.2
Too active	89	7.2	9	3.2	7	6.5	2	3.7	96	7.2	11	3.3
Too noisy	4	0.3	4	1.4	–	–	–	–	4	0.3	4	1.2
Unable to train	4	0.3	–	–	–	–	–	–	4	0.3	–	–
**Owner circumstances**	122	9.9	32	11.3	5	4.6	5	9.3	127	9.5	37	10.9
Cannot afford	3	0.2	3	1.1	–	–	–	–	3	0.2	3	0.9
Change in lifestyle	16	1.3	4	1.4	–	–	–	–	16	1.2	4	1.2
Divorce/separation	1	0.1	1	0.4	–	–	–	–	1	0.1	1	0.3
New baby	4	0.3	–	–	–	–	–	–	4	0.3	–	–
Not enough time	53	4.3	11	3.9	3	2.8	–	–	56	4.2	11	3.3
Personal problems	41	3.3	12	4.2	2	1.9	4	7.4	43	3.2	16	4.7
Travel	4	0.3	1	0.4	–	–	1	1.9	4	0.3	2	0.6
**Health of owner**	65	5.3	58	20.4	2	1.9	2	3.7	67	5.0	60	17.8
Allergic to animal	44	3.6	53	18.7	1	0.9	2	3.7	45	3.4	55	16.3
Death of owner/family	1	0.1	–	–	–	–	–	–	1	0.1	–	–
Health of owner/family	20	1.6	5	1.8	1	0.9	–	–	21	1.6	5	1.5
**Health of animal**	29	2.4	16	5.6	11	10.2	17	31.5	40	3.0	33	9.8
**Housing**	143	11.6	11	3.9	8	7.4	–	–	151	11.3	11	3.3
Inadequate housing/yard	23	1.9	1	0.4	1	0.9	–	–	24	1.8	1	0.3
Landlord issues	93	7.6	5	1.8	6	5.6	–	–	99	7.4	5	1.5
No home	2	0.2	1	0.4	1	0.9	–	–	3	0.2	1	0.3
Military transfer of owner	2	0.2	1	0.4	–	–	–	–	2	0.1	1	0.3
Moving	23	1.9	3	1.1	–	–	–	–	23	1.7	3	0.9
**Not compatible with children**	21	1.7	6	2.1	4	3.7	1	1.9	25	1.9	7	2.1
**Not compatible with pets**	247	20.1	72	25.4	18	16.7	10	18.5	265	19.8	82	24.3
Doesn't like other pets	131	10.7	29	10.2	12	11.1	7	13.0	143	10.7	36	10.7
Pets in home didn't like	116	9.4	43	15.1	6	5.6	3	5.6	122	9.1	46	13.6
**Not compatible with owner**	50	4.1	6	2.1	7	6.5	–	–	57	4.3	6	1.8
Litterbox odor	–	–	1	0.4	–	–	–	–	–	–	1	0.3
Sheds	1	0.1	–	–	–	–	–	–	1	0.1	–	–
Too big	11	0.9	–	–	2	1.9	–	–	13	1.0	–	–
Too much responsibility	37	3.0	5	1.8	5	4.6	–	–	42	3.1	5	1.5
Wrong sex	1	0.1	–	–	–	–	–	–	1	0.1	–	–
**Other**	2	0.2	–	–	–	–	–	–	2	0.1	–	–
Abandoned by owner	1	0.1	–	–	–	–	–	–	1	0.1	–	–
Insurance restrictions	1	0.1	–	–	–	–	–	–	1	0.1	–	–
**Unrealistic expectations**	99	8.1	25	8.8	6	5.6	1	1.9	105	7.9	26	7.7
**Unwanted**	14	1.1	4	1.4	2	1.9	–	–	16	1.2	4	1.2
**Total**	1229	100.0	284	100.0	108	100.0	54	100.0	1337	100.0	338	100.0

### Post-return adoptions

**Figure 2:**
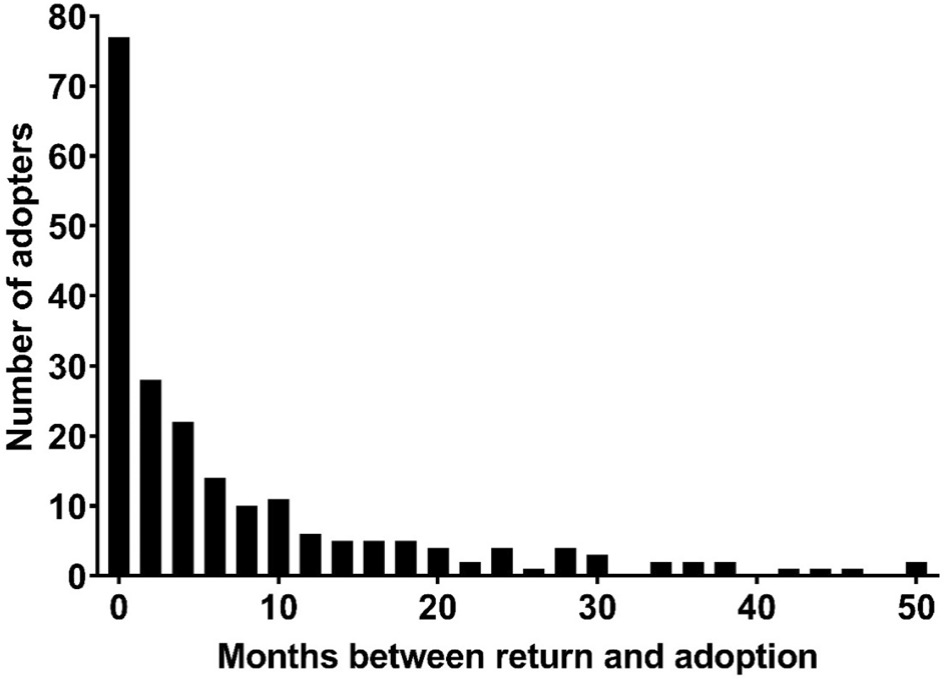
Months between return and adoption post-return for all owners who adopted a new animal during the study period (*n* = 210).

10.5% of return owners adopted a new animal following return, including 144 individuals who returned dogs, 65 individuals who returned cats and one individual who returned a rabbit. The median length of time between return and post-adoption return was 3.2 months (Fig. 2), with no difference between cat and dog returns (*t*(207) = 1.24, *p* = 0.22). The adopted animals included 107 dogs, 101 cats, one rabbit, and one guinea pig.

A binary logistic regression model revealed the likelihood of post-return adoption within 12 months was associated with species (including cats and dogs only). Individuals who returned cats were 2.45 times more likely to adopt a new animal compared with individuals who returned dogs (OR 2.45, 95% CI 1.62, 3.72). The reasons for return were also associated with the likelihood of post-return adoption. Owners who returned animals due to the animal’s health were four times more likely to adopt a new animal compared with owners who returned animals for behavioral reasons (OR 4.20, 95% CI 2.37, 7.45). Owners who returned animals due to their personal health or circumstances were 80% and 59% less likely to adopt another animal compared with owners who returned animals for behavioral reasons (OR 0.20, 95% CI 0.07, 0.57 and OR 0.41, 95% CI 0.20, 0.82). ‘Unrealistic expectations’ and ‘not compatible with pets’ were also associated with decreased odds of post-return adoption compared with returns due to behavioral reasons (OR 0.42, 95% CI 0.19, 0.94, and OR 0.61, 95% CI 0.38, 0.98). Returns due to incompatibility with the owner or children, housing issues, or ‘unwanted’ were not associated with the likelihood of post-return adoption (*p*  ≥0.14). Length of stay in the home (*p* = 0.89) and the sex (*p* = 0.88) of the returned animal did not predict the odds of post-return adoption. The likelihood of post-return adoption was also not significantly associated with the returned animal’s age group, although there was a trend towards an increased likelihood of post-return adoption among adult animals (OR 1.61, 95% CI 0.95, 2.72, *p* = 0.08).

### Characteristics of post-return adoptions

Most owners adopted the same species post-return (75.7%, *n* = 159), but 24.3% adopted a different species (*n* = 51). Of those who adopted a different species, 84.3% adopted a dog initially and then adopted a cat post-return (*n* = 43), whereas 13.7% adopted a cat initially and then adopted a dog post-return (*n* = 7). One individual returned a dog and then adopted a rabbit. Considering individuals who adopted the same species post-return, almost half adopted an animal of a different sex post-return (43.4%, *n* = 69), although the direction of change was split. Thirty-two owners returned a female and then adopted a male animal post-return, and 37 owners returned a male and then adopted a female post-return. Most returning owners adopted from the same age group post-return (84.8%, *n* = 178). All owners who adopted an animal of a different age group chose an older animal post-return (*n* = 32).

## Discussion

To date, the impact of unsuccessful animal adoptions on the likelihood of owners adopting a new animal post-return has not been established. At this large animal shelter in the Southeastern United States, one in 10 individuals adopted a new animal from the shelter post-return suggesting the unsuccessful adoption experience decreased adopters’ desire to acquire another pet from the shelter. The majority of individuals who adopted again did not change their animal preferences post-return with the exception of sex, whereby half of returning owners adopted an animal of a different sex following return. We also found one quarter of individuals adopted a different species following return, most of whom returned a dog and adopted a cat. It is possible that individuals who returned dogs were more inclined to adopt cats post-return due to the lower perceived costs and responsibilities of cat ownership compared with dog ownership^21^.

The reasons for return had a significant influence on the likelihood of future adoptions. Owners who returned animals due to the animal’s health were four times more likely to adopt post-return compared with owners who returned animals for behavioral issues. Behavior problems have been associated with greater ownership costs^22–24^, decreased ownership satisfaction^23^, decreased human-animal attachment^25^ and poorer mental wellbeing among owners^24–26^. In contrast, compatibility between owners and their pets on key behavioral characteristics, such as enjoying exercise and getting along with peers, has been associated with greater ownership satisfaction and happiness, and decreased stress^27,28^. Research also indicates that caring for an animal with medical needs can increase stress and anxiety and reduce owners’ quality of life^29–31^. It is therefore interesting that returns due to animal behavior had such a detrimental effect on the likelihood of future adoptions compared with medical concerns. It seems a mismatch between the animal’s behavioral needs and the owner’s willingness to tolerate behavioral issues could damage the adopter’s long-term relationship with the shelter. Considering that behavior is a leading cause of post-adoption returns^6,7,11,12^, it is imperative that animal shelters aim to minimize behavioral incompatibility between adopters and their animals. The efficacy of adoption counselling and adopter-animal matching programs in reducing returns is a developing area of research that warrants further attention. Preliminary evidence suggests some policies, such as only showing adopters the animals that match their needs, may be associated with reduced return rates but additional research is needed^32^.

Individuals who returned animals for owner-related reasons were considerably less likely to adopt post-return. For example, owners who returned animals due to their health or the health of their family were 80% less likely to adopt again. This finding is logical as health concerns that are exacerbated by pet ownership, such as allergies, may not improve with the introduction of a different pet. Previous research has found owners who relinquished animals due to allergies often viewed their situation as insurmountable^19^. Returns due to owners’ circumstances were also associated with a 60% reduction in the odds of future adoptions. Again, owners’ circumstances, such as personal problems or a lack of time, may not improve with the introduction of a different pet.

Returns due to unrealistic expectations for ownership were associated with a 60% reduction in the likelihood of future adoptions. Ownership satisfaction has been shown to decrease with greater perceived costs of ownership, including lifestyle, time, and financial costs^23^. Adopters who underestimated the effort involved in caring for an animal may have been dissatisfied with pet ownership and therefore, less motivated to adopt again post-return. It is also possible that some owners had unrealistic expectations for benefits attributable to pet ownership. Previous research indicates that individuals with dog ownership history (previous or current owners) are more likely to expect mental and psychosocial health benefits than prospective owners with no prior experience, possibly due to bias arising from their affection towards their previous/current dog^33^. Data regarding the influence of previous ownership history on the risk of returns is mixed. One study found adopters with previous ownership experience returned animals more frequently due to behavioral issues than first-time owners^6^, but other studies have found first-time owners are more likely to return animals^9^. A lack of data regarding adopters’ lifetime pet ownership history precluded us from investigating the role of previous pet ownership on post-return adoptions in the current study. Incompatibility with existing pets was also associated with 40% lower odds of post-return adoption, suggesting that some owners concluded they did not want to upset the current pet dynamic or that their current pet was better suited as the only pet in the household.

Individuals who returned cats were two and a half times more likely to adopt post-return compared with dog adopters. This finding may be attributable to the differences in return reasons between dogs and cats, such as the higher rate of cat returns due to the animal’s health. Cat and dog adopters may also differ in their expectations for ownership which could affect the likelihood of owners adopting post-return^9,34,35^. For instance, preliminary data suggests cat owners believe their ability to control or modify their cat’s behavior is low^36,37^, while most dog owners anticipate the need for training and expect to encounter some difficulties with dog behavior^33^. Cat adopters that experience undesirable behavior may believe the behavior cannot be modified and that a different cat would be better suited to their household. Alternatively, dog adopters that face difficulties with behavior may feel it is their responsibility to work with the dog to modify its behavior. If the behavior is too challenging for the adopter, resulting in the animal’s return to the shelter, the owner may question whether they have the time or resources necessary for dog ownership. It is also possible that individuals who returned dogs to the shelter may have acquired a dog from a different source post-return. Future studies might focus on the differences in adopters’ expectations of ownership based on species and their role in post-return adoptions.

Length of stay in the home was not associated with the odds of future adoptions. The mean length of ownership in this study was relatively short, and it is possible that the provision of adoption vouchers for returns within 30 days incentivized adopters to return the animal within this period. However, this does not explain the significant number of returns that occurred within the first week of ownership. It is also plausible that adopters observed the problem that led to return relatively quickly after bringing the animal home. Previous work has found half of returning adopters observed the problem that led to return immediately after adoption, and a further 17% observed the problem within the first week^20^. The short length of ownership may have limited differences in the strength of the human-animal bond, perhaps reducing any impact of length of ownership on the likelihood of readoption. We also found the sex of the returned animal was not associated with the likelihood of post-return adoption.

The findings presented in this study are subject to several limitations. Firstly, the data reflect a single animal shelter and research across multiple facilities is needed to confirm our findings. The retrospective nature of the study also necessitates cautious interpretation. For example, we could not establish how many owners, if any, had acquired pets through alternative sources, so the true rate of pet acquisition following unsuccessful animal adoptions may be significantly higher than suggested here. Nevertheless, our findings speak to the importance of the unsuccessful adoption experience on individuals’ willingness to adopt from the same animal shelter and indicate potential long-term effects of returns on the shelter-adopter relationship. The retrospective design also resulted in a reliance on owner-reported return reasons which may be subject to bias or inaccuracies^38,39^. For example, returns due to behavioral issues are likely affected by the owner’s understanding of animal behavior, and previous research suggests owners’ ability to recognize animal behavior is poor^40–42^. However, given that this study focused on the owner-related effects of unsuccessful animal adoptions, the owner’s perceived return reason is important irrespective of the accuracy of the reason itself.

## Conclusion

At this large animal shelter in the Southeastern United States, one in 10 returning owners acquired an animal from the shelter following return. The likelihood of post-return adoption was associated with the reasons for return and species. Individuals who returned animals for owner-related reasons were significantly less likely to adopt post-return compared with owners who returned due to animal-related reasons. However, owners who returned animals due to the animal’s health were four times more likely to adopt post-return compared with those who returned them for behavioral reasons. The experience of an unsuccessful animal adoption appears to suppress an individual’s desire to adopt again, particularly when returns occur due to owner-related reasons or animal behavior. Future, prospective studies might investigate the use of alternative acquisition sources post-return and elucidate possible differences in the experiences of people who adopt again and those who do not.

## Methods

### Shelter characteristics

Charleston Animal Society is a large, open admission animal shelter in South Carolina, United States. Between 2015 and 2019, the shelter’s total live intake included 17,664 dogs and 23,525 cats. Most animals entered the shelter as strays, including 72% of dogs and 89% of cats. Charleston Animal Society employs an open adoption policy that aims to create a trusting and communicative relationship with adopters. The shelter encourages all adopters to return their animal/s to the shelter if necessary and provides a refund voucher for future adoptions if the animal is returned within 30 days (excluding animals adopted during fee-waived promotions). Charleston Animal Society also offers post-adoption support in the form of a free veterinary appointment at a local veterinary clinic and free behavioral support. The shelter’s behavior team conduct follow-up calls for animals with known behavioral problems in the shelter where possible, and adopters have the opportunity to contact the shelter to request behavioral advice and support if needed. The study was determined exempt from review by the University of Pennsylvania Institutional Review Board (protocol number 84837). The study was carried out in accordance with relevant guidelines and regulations.

### Data records and variables

Data from individuals who adopted and returned an animal to the Charleston Animal Society between 1st January 2015 and 31st December 2019 were included in the study (*n* = 2073). Cases were excluded if the person’s ID number differed between the adoption and the return (*n* = 74). Data were downloaded from the shelter’s electronic records (PetPoint Data Management System, Version 5, Pethealth Software Solutions Inc., USA) and the following variables were extracted: animal species, sex, known/estimated date of birth, adoption date/s, return date/s and reason/s for return.

Animal shelter staff recorded a single return reason in PetPoint at the time of return. Researchers then categorized the return reasons into the following groups: behavior, owner circumstances, health of owner, health of animal, housing, not compatible with children, not compatible with pets, not compatible with owner, unrealistic expectations, unwanted and other (Table 1).

The animal’s age at adoption was calculated as the number of months between the known/estimated date of birth and the date of adoption. Adoption age was then categorized as puppy/kitten (< 6 months), young adult (> 6 months–2 years), adult (> 2–8 years) and senior (> 8 years). Length of stay in the home was calculated as the number of days between the adoption date and the return date. For owners who adopted post-return, the time between return and post-return adoption was calculated as the number of days between the return date and the second animal’s adoption date. A binary variable was created to compare individuals who adopted within 12 months of return and those who did not adopt within 12 months. Twelve months was chosen as the cut-off value as this captured most individuals who adopted post-return while excluding outliers that may have adopted years later.

### Statistical analysis

All statistical analyses were conducted in IBM SPSS Statistics for Windows, version 24. A Pearson’s Chi-Square test was used to compare return reasons, and an independent t-test was used to compare the time between return and post-return adoption between dogs and cats. A binary logistic regression model was used to investigate the associations between post-return adoption (within 12 months) and the reasons for return, species (cats and dogs only), sex, length of stay in the home and age group of the returned animal. Returns due to ‘insurance restrictions’ (*n* = 1) or ‘abandoned by owner’ (*n* = 1) were excluded from the model due to the low number of cases. Statistical significance was set at p < 0.05.

## Data Availability

The data governance arrangements for the study do not allow us to redistribute Charleston Animal Society data to other parties.
